# Resolvin D1 suppresses pannus formation via decreasing connective tissue growth factor caused by upregulation of miRNA-146a-5p in rheumatoid arthritis

**DOI:** 10.1186/s13075-020-2133-2

**Published:** 2020-03-27

**Authors:** Weiwei Sun, Jinglan Ma, Han Zhao, Chipeng Xiao, Hao Zhong, Hanzhi Ling, Zhen Xie, Qingqing Tian, Huaijun Chen, Tingting Zhang, Mu Chen, Shengwei Jin, Jianguang Wang

**Affiliations:** 1 Department of Biochemistry, School of Basic Medical Sciences, Wenzhou Medical University, Wenzhou, Zhejiang Province China; 2 Department of Anesthesia and Critical Care, the Second Affiliated Hospital of Wenzhou Medical University, Wenzhou, 325027 Zhejiang Province China

**Keywords:** Rheumatoid arthritis, Resolvin D1, MiRNA-146a-5p, Connective tissue growth factor, Inflammatory mediators, Pannus

## Abstract

**Background:**

Rheumatoid arthritis (RA) is a chronic autoimmune disease characterized by inflammation and joint stiffness, finally leading to tissue destruction. Connective tissue growth factor (CTGF) is a critical factor in RA progression, which promotes fibroblast-like synoviocyte (FLS) proliferation, pannus formation, and the damage of cartilage as well as bone. Resolvin D1 (RvD1) can promote inflammation resolution in acute inflammatory diseases, and recently, effects of RvD1 on chronic inflammatory diseases also attracted attention. This study aimed to examine the effect of RvD1 on pannus formation in RA and the underlying mechanism.

**Methods:**

Serum levels of RvD1 and CTGF were determined in RA patients and healthy persons by UPLC-MS/MS and ELISA respectively. The levels of CTGF and inflammatory factors were assessed by qRT-PCR and ELISA. MicroRNA expression profile was determined by miRNA microarray. The effects of CTGF, RvD1, and miR-146a-5p on angiogenesis were evaluated with tube formation and chick chorioallantoic membrane (CAM) assays. Collagen-induced arthritis (CIA) mice were constructed to detect the effects of RvD1 and miR146a-5p on RA. STAT3 activation was determined by Western blotting.

**Results:**

RvD1 levels decreased while CTGF levels increased in RA patients’ serum, and an inverse correlation of the concentrations of RvD1 and CTGF in the serum of RA patients was synchronously observed. In CIA mice, RvD1 suppressed angiopoiesis and decreased the expression of CTGF. Simultaneously, RvD1 significantly decreased CTGF and pro-inflammation cytokines levels in RA FLS. Furthermore, CTGF suppressed angiopoiesis and RvD1 inhibited the proliferation and migration of RA FLS and angiopoiesis. MiRNA microarray and qRT-PCR results showed that RvD1 upregulated miRNA-146a-5p. The transfection experiments demonstrated that miRNA-146a-5p could decrease inflammatory factors and CTGF levels. Moreover, miRNA-146a-5p decreased the proliferation of FLS and angiogenesis in vivo. MiRNA-146a-5p also suppressed angiogenesis and downregulated the expression of CTGF in CIA mice. Finally, Western blot results revealed that miRNA-146a-5p inhibited the activation of STAT3.

**Conclusion:**

RvD1 is prone to alleviate RA progression through the upregulation of miRNA-146a-5p to suppress the expression of CTGF and inflammatory mediators, thereby decreasing pannus formation and cartilage damage.

**Electronic supplementary material:**

The online version of this article (10.1186/s13075-020-2133-2) contains supplementary material, which is available to authorized users.

## Background

Rheumatoid arthritis (RA) is a chronic autoimmune disease characterized by inflammation, joint stiffness, and pain, finally leading to tissue destruction [1]. Excessive proliferation fibroblast-like synoviocytes (FLS) play a vital role in RA progression, which promotes pannus formation, produces inflammatory mediators, and finally aggravates cartilage damage [2]. Pannus formation is one of the driving pathologic processes which can lead to the development of joint erosion in RA. Connective tissue growth factor (CTGF) is a cysteine-rich protein secreted by FLS in RA patients, which can induce the proliferation of FLS to form pannus, attack cartilage, and exacerbate the disease [3]. Our previous proteomic research showed that CTGF expression in FLS from RA patients was obviously higher than that in the controls [4], and the clinical results revealed that the concentration of CTGF in serum of RA patients showed the same tendency. Besides, CTGF was a biomarker for the diagnosis of RA [5]. Furthermore, it was also demonstrated that CTGF can prompt human umbilical vein endothelial cell (HUVEC) proliferation and migration [4]. These results indicate that CTGF correlates well with RA disease activity. Nozawa et al. found that anti-CTGF antibody could reduce the clinical score and the content of IL-2 and matrix metalloproteinase-3 in serum in collagen-induced arthritis (CIA) mice [6]. The findings hint that the drug which can regulate the expression of CTGF may effectively ameliorate disease progression in patients with RA.

Resolvins, one of specialized pro-resolving mediators (SPMs), are derived from omega-3 fatty acids during the resolution phase of inflammatory response. Resolvins include E resolvins (RvE) and D resolvins (RvD), and RvD comprises RvD1–6 [7]. They have been reported to have many functions, including limiting the migration of neutrophils [8–10], suppressing the production of inflammatory factors, and strengthening phagocytic ability of macrophages in acute inflammation [11]. The effects of resolvins on chronic inflammatory disease have also attracted attention in recent years. Arnardottir et al. found that resolvin D3 (RvD3) could reduce the number of leukocytes in serum to ameliorate RA progression [12]. Besides, Lima-Garcia et al. also discovered that resolvin D1 (RvD1) could relieve pain in adjuvant-induced arthritis in rats [13]. These findings suggest that RvD1 is associated with RA. However, whether or not RvD1 can inhibit the expression of CTGF and pannus formation in RA progression is still unclear.

MicroRNAs (miRNAs) are a family of small non-coding RNAs that usually downregulate gene expression through targeting the 3′-UTR of mRNA [14]. Recently, there are increasing researches revealing that miRNAs play an important role in RA progression [15]. In FLS of RA patients, miRNA-23b can regulate arthritic inflammation by inhibiting the NF-κB signal pathway [16]. Furthermore, SPMs can regulate the Treg/Th17 imbalance in CIA mice by upregulating miR-21 [17]. These studies indicate that RvD1 is apt to exert its biological functions through microRNA.

In this study, we aimed to determine the effect of RvD1 on pannus formation and the expression of CTGF in RA progression, and shed light on the function of microRNA during this process.

## Materials and methods

### RvD1

RvD1 (C22H32O5, 7S,8R, 17S-trihydroxy-4Z, 9E, 11E, 13Z, 15E, 19Z-docosahexaenoicacid, CAS No. 872993–05-0) was purchased from Cayman Chemical Company, Ann Arbor, USA (cat. Number 10012554). The concentration and purity of the RvD1 were identified by UPLC-MS/MS. RvD1 was aliquoted into several brown glass tubes by a glass HAMILTON syringe to guarantee the activity. The tubes were then evacuated oxygen by nitrogen gas and were stored at − 80 °C to avoid repeated freezing and thawing.

### Patients and samples

Samples were acquired from RA patients and healthy controls at the First Affiliated Hospital of Wenzhou Medical University from May 2016 to May 2017. RA diagnosis was in accordance with the 2010 American College of Rheumatology (ACR) criteria. The clinical information of the patients was shown in supplementary Table 1. Serum was obtained from patients with RA on the first day of clinical admission before undergoing any treatment, and the control serum was from the healthy individuals. This study was approved by the Clinical Research Ethics Committees of the First Affiliated Hospital of Wenzhou Medical University (No. 2016157). All patients participated in this study provided written informed consent.

### The concentration of RvD1 determined by UPLC-MS/MS

The concentration of RvD1 was measured as described previously [18]. Each sample was dissolved with ice-cold methanol. RvD1 levels were determined by ultra-performance liquid chromatography tandem mass spectrometry (UPLC-MS/MS) using an UPLC I-Class system (Waters, USA) equipped with an Agilent Eclipse Plus C18 column (2.1 mm × 100 mm × 1.7 μm) paired with Sciex 6500 Q-TRAP mass spectrometer (Sciex, USA). To monitor and quantify the levels of the various LM, a multiple reaction monitoring (MRM) method was developed with signature ion fragments for RvD1. Identification and quantification of RvD1 were conducted using previously published criteria in which a minimum of 6 diagnostic ions were employed and based on peak area of MRM transitions and the linear calibration curve of RvD1 respectively.

### Collagen-induced arthritis (CIA) model

DBA/1 mice (male, 7–8 weeks old) were obtained from SLAC Laboratory Animal Co. (Shanghai, China). All experiment procedures were approved by the Institutional Animal Care and Use Committee of Wenzhou Medical University. CIA model in DBA/1 mice was induced as described previously [19]. Briefly, on day 0, mice were injected intradermally at the base of the tail with 100 μL of type II bovine collagen (2 mg/mL, Chondrex, USA) emulsified in equal volumes of complete Freund’s adjuvant (CFA, Sigma-Aldrich, USA) containing heat inactivated Bacillus Calmette-Guerin. After 21 days, mice were given a booster immunization with 100 μL of type II bovine collagen (2 mg/mL) emulsified in equal volumes of incomplete Freund’s adjuvant (IFA, Sigma-Aldrich, USA). On the booster immunization day, mice were treated with RvD1 (0, 20, and 100 ng) by tail vein injection every third day or mouse miRNA146a-5p agomir by intra-articular injection once a week until day 48. From day 21 to day 48, the mean clinical score of each mouse was evaluated according to the standardized method [19]. The mean clinical score (0–4) was assigned as follows: 0 = no symptoms, 1 = erythema and slight swelling limited to the ankle joint and toes, 2 = erythema and slight swelling spreading from the ankle to the midfoot, 3 = erythema and severe swelling spreading from the ankle to the metatarsal joints, and 4 = ankylosing deformity with joint swelling. Mice were sacrificed on day 49. To collect the synovial fluid from mice, saline was injected into mice articular cavity, and then gathered. Serum and joint tissues were harvested for further study.

### Histopathology evaluation

Samples were obtained from the knee joints of sacrificed mice. Then the specimens were fixed in 4% paraformaldehyde, decalcified in 50 nM EDTA, and embedded in paraffin. They were afterwards serially sectioned onto microscope slides at a thickness of 5 μm and then deparaffinized, rehydrated, and stained with hematoxylin and eosin (H&E) or toluidine blue.

### ELISA

The concentrations of TNF-α, IL-1β, and IL-6 in mice serum and CTGF in human serum were detected using specific commercially available ELISA kits according to the manufacturer’s instructions (GenWay Biotech, USA). The concentrations of TNF-α, IL-1β, IL-6, and CTGF in supernatants from RA FLS were determined by commercially available ELISA kits (GenWay Biotech, USA). The concentration of CTGF in mice serum was detected by commercial ELISA kit according to the manufacturer’s instructions (Biorbyt, England).

### Isolation and culture of RA FLS

RA FLS were isolated from synovial tissues according to the method described previously [20]. Passage 3–5 RA FLS were used for each experiment, for these, cells were purer and had better biological functions than other passages. This study was approved by the Clinical Research Ethics Committees of the First Affiliated Hospital of Wenzhou Medical University (No. 2016157). All RA patients signed informed consent before the research started.

### MTT assay

A 3-(4.5-dimethylthiazol-2-yl)-2,5-diphenyltetrazolium bromide (MTT) assay was used to assess the effects of RvD1 and miRNA-146a-5p on RA FLS proliferation. The RA FLS were seeded in a 96-well plate at the concentration of 1 × 10^6^/ml and incubated for 24 h. Then the cells were treated with various concentrations of RvD1 (0, 20, and 100 nM) or human miRNA-146a-5p mimics. The experiments were performed in duplicate. Plates were incubated at 37 °C in 5% CO_2_ for different times (12 h, 24 h, 48 h, and 72 h), and MTT dissolved with dimethyl sulfoxide (DMSO; Sigma-Aldrich, USA) was added to the culture medium 4 h before the final test. The absorbance value at the 490 nm wavelength was measured using a Biotek Synergy2 spectrophotometer (Winooski, VT, USA).

### Scratch migration assay

RA FLS were seeded in six-well plates at a density of 2.5 × 10^5^ cells/well. A scratch was made along the diameter of the well using a 10-μL pipette tip (Axygen® Corning, USA) once the cells reached close to 95% confluency. The wells were gently washed 3 times with PBS to remove the detached cells. DMEM containing various concentrations of RvD1 (0, 20, 100 nM) was added to the wells, and the cells were grown for an additional 72 h. During this period, images were captured after 24 h, 48 h, and 72 h incubation by a camera under the light microscope (× 40 magnification). ImageJ 2.43 s was used to calculate the mobility ratio, and the mobility ratio was calculated using the following equation: migrated cellular area/scratched area × 100%.

### Endothelial tube formation assay

Endothelial tube formation assay was performed to evaluate the angiogenic activity in vitro as described previously [21]. Matrigel was diluted with DMEM at the ratio of 1:1, then 96-well plates were coated with Matrigel and incubated at 37 °C for about 1 h to promote gelling. Human umbilical vein endothelial cells (HUVEC) were added to each well with different concentrations of RvD1 (0, 20, 100 nM), human miRNA-146a-5p agomir, or human CTGF-specific siRNA lentivirus (Genepharma, China) and incubated in a humidified incubator at 37 °C with 5% CO_2_. After 4 h, 6 h, and 8 h incubation, the plates were observed and captured by a camera under a microscope. Tube-like structures in each well were evaluated by the number of intersections among branches of the endothelial cell networks in the whole field.

### CAM assay

The chick chorioallantoic membrane (CAM) assay was carried out to evaluate the angiogenic activity in vivo as described previously [22]. Embryonated chicken eggs (~ 10 per treatment) were incubated at 37 °C and treated with RvD1 (0, 20, 100 nM), chicken miRNA-146a-5p agomir or a lentiviral vector harboring RNAi sequence targeting the CTGF gene (Gene Pharma, China) respectively, which were absorbed on sterile Whatman GB/B glass fiber filter disks (6 mm in diameter; Reeve-Angel, Clifton, NJ, USA) on day 7. The blood vessels in embryos were observed 3 days later under a stereomicroscope. ImageJ 2.43 s was used to assess the vascular and CAM areas. The percentage of angiogenic area was calculated using the following equation: vascular area / CAM area × 100%.

### MiRNA microarray and MiRNA transfection assay

The microRNA expression profiling of RA FLS treated with RvD1 was determined by miRNA microarray analysis with the human miRNA array probes (Exiqon, Denmark). QRT-PCR was used to validate the upregulation of miRNA-146a-5p. RA FLS were then transfected with human miRNA146a-5p mimic/inhibitor or a non-sense strand negative control using Lipofectamine™ 3000 as a transfection reagent. Lipofectamine 3000 was mixed with 50 μl Opti-MEM. Meanwhile, miRNA146a-5p mimic/inhibitor was mixed with 50 μl Opti-MEM. The two mixtures were then mixed for 5 min and then added to the cell culture medium and left to incubate for 48–72 h at 37 °C in 5% CO_2_. Cells were collected for total RNA extraction.

### Quantitative real-time PCR analysis

Total RNA samples in RA FLS were isolated by TRIzol Reagent according to the manufacturer’s protocol. The cDNA was synthesized by PrimeScript™RT reagent Kit with gDNA Eraser (TAKARA, Japan) and the expression of mRNA was detected with SYBR® Premix Ex Taq™ II (TAKARA, Japan) by quantitative real-time PCR (qRT-PCR). qRT-PCR of miRNA was performed with miRNA First-Strand Synthesis and SYBR qRT-PCR kit (Clontech, USA). The gene-specific primers used were listed in supplementary Table 2. The relative expression of miRNA was normalized to U6 controls while mRNA was normalized to β-actin and was calculated using the 2^−ΔΔCt^ method.

### Western blot analysis

Western blot analysis from RA FLS homogenates were performed as described previously [23]. Equal amounts of protein per sample were separated by 10% SDS-PAGE, and subsequently transferred to nitrocellulose membrane (Pierce). The membranes were blocked for 2 h with 5% skimmed milk and then incubated in the primary antibodies overnight at 4 °C. Horseradish peroxidase-linked anti-rabbit antibodies were used as secondary antibodies. The membranes were imaged with the Image Quant LAS 4000 mini (GE Healthcare Bio-Sciences AB, Uppsala, Sweden).

### Statistical analysis

SPSS software 19.0 and GraphPad Prism 7.0 were employed to analyze the data. All results were expressed as means ± SD. All of the experiments were carried out in triplicate. Normally distribution of data was determined by the Shapiro-Wilk method, and the homogeneity of variance was determined by Levene method. Student’s *t* test was used to analyze the difference between two sets of data that met the normal distribution and homogeneity of variance. One-way analysis of variance test was applied to analyze the differences among multigroups.

## Results

### RvD1 levels decreased while CTGF levels increased in serum of RA patients

**Fig. 1:**
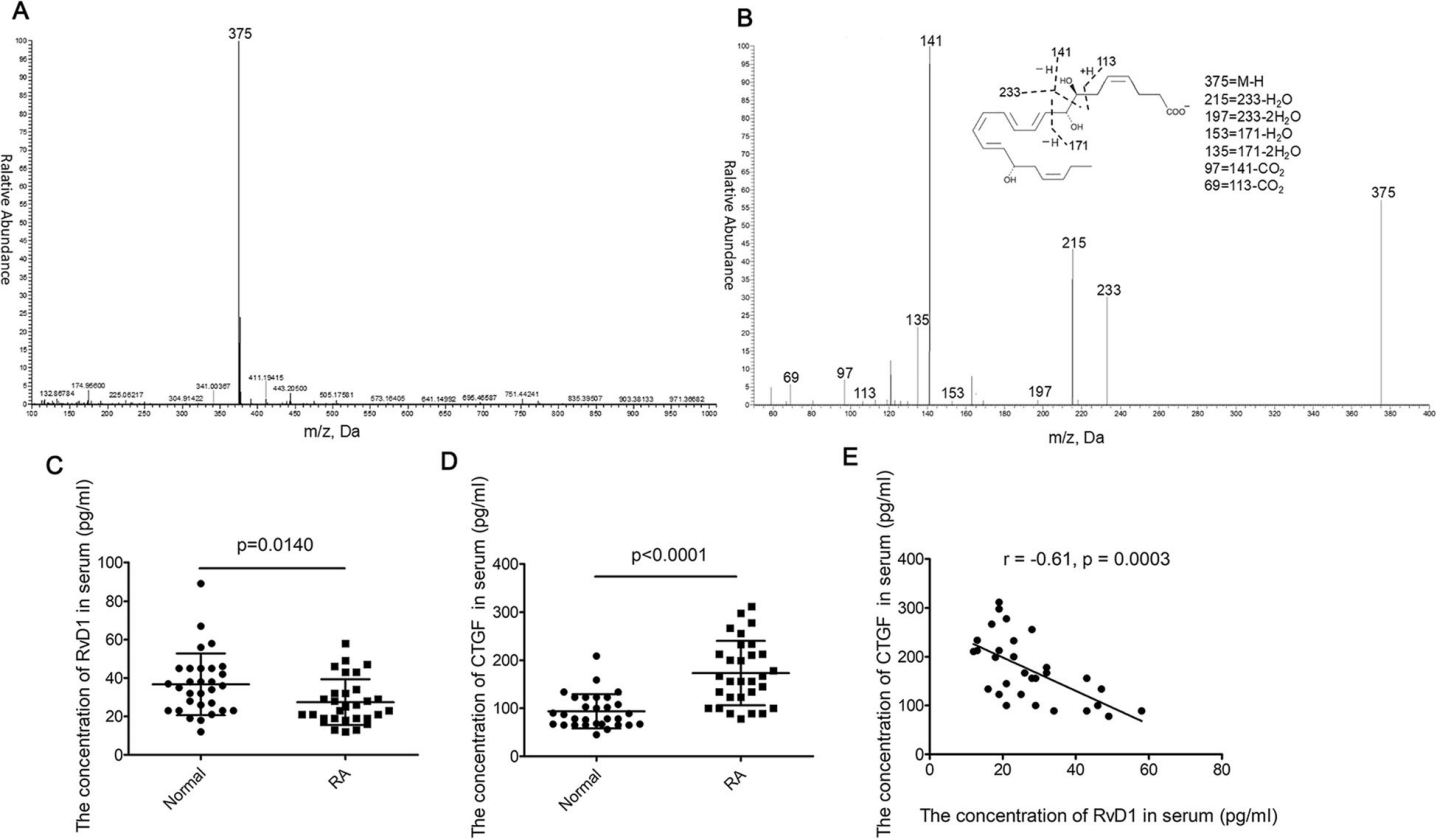
RvD1 levels decreased, while CTGF levels increased in serum of RA patients. RvD1 was determined by UPLC-MS/MS, and CTGF was examined by ELISA kit. **a** Multiple reaction monitoring (MRM) chromatogram for RvD1 (m/z = 375/141). Q1, M-H (parent ion); Q3, diagnostic ion in the tandem mass spectrometry (MS/MS) (daughter ion). **b** MS/MS spectrum of RvD1 in serum. **c** The concentration of RvD1 in serum of RA patients (*n* = 30) was lower than that of the normal persons (*n* = 30). **d** The serum CTGF level from RA patients (*n* = 30) was significantly increased compared to that from the health controls (*n* = 30). **e** Pearson coefficient analysis was used to determine the linear correlation between the concentrations of CTGF and RvD1 in serum (*r* = − 0.61, *p* = 0.0003). All data was represented as the mean ± SD. Student’s *t* test was used to evaluate the statistical significance between the normal and RA group

The concentrations of CTGF and RvD1 in RA patients and the health controls were determined. As depicted in Fig. 1, serum RvD1 levels in RA patient were 26.34 pg/ml (95% confidence interval [CI] 22.55–30.14 pg/ml), which were significantly lower than those in normal persons (38.09 pg/ml, 95% CI 32.69–43.48 pg/ml) (Fig. 1c). On the contrary, serum CTGF levels in RA patients (123.5 pg/ml, 95%CI 94.08–152.84 pg/ml) were remarkably higher than those in normal persons (81.14 pg/ml, 95% CI 68.24–94.04 pg/ml) (Fig. 1d). Furthermore, an inverse correlation of the concentrations of CTGF and RvD1 in serum was observed with *r* = − 0.61, *p* = 0.0003 (Fig. 1e).

### RvD1 attenuated joints damage and inflammatory response in CIA mice

**Fig. 2:**
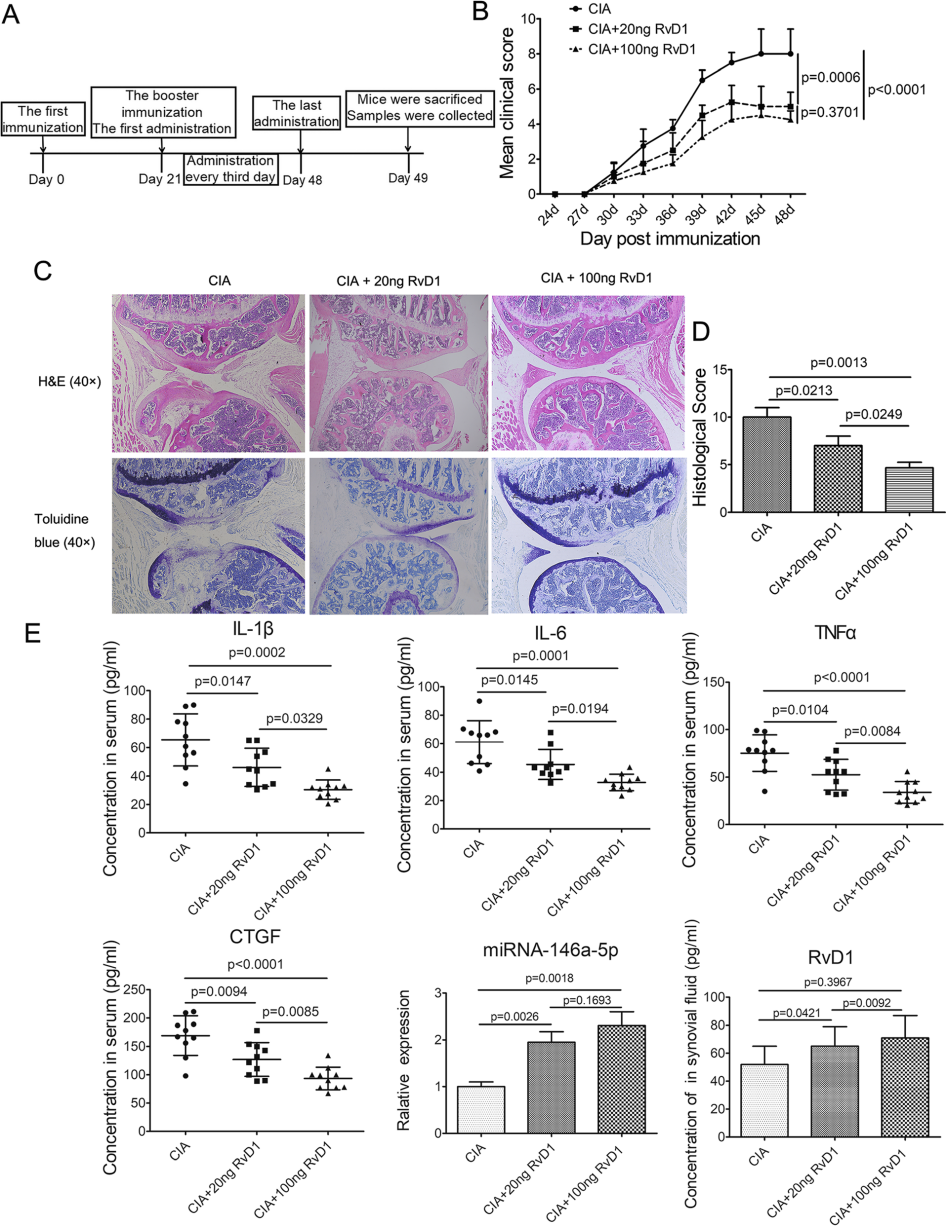
RvD1 alleviated joint injury and inflammatory response in the CIA mice. **a** The timeline of the RvD1-treated CIA mice experiment. **b** The mean clinical score of CIA mice. Analysis of variance (ANOVA) of repeated measurement was used to analyze the statistical significance. **c** H&E and toluidine blue staining of the knee joints of the mice (left panel). **d** Histological scores (synovial infiltration, synovial hyperplasia, bone destruction, and cartilage damage were assessed) were graded on a scale of 0 (normal) to 3 (severe) in 12 totally (right panel). **e** Pro-inflammatory cytokines (TNF-α, IL-1β and IL-6) and CTGF in serum were measured by ELISA. The expressions of miRNA-146a-5p in joints from mice were determined by Q-PCR. The concentration of RvD1 in synovial fluid from CIA mice was measure by UPLC-MS/MS. CIA, CIA mice were intravenously injected with PBS; CIA + 20 ng RvD1, CIA mice were intravenously injected with 20 ng RvD1 per mouse; CIA + 100 ng RvD1, CIA mice were intravenously injected with 100 ng RvD1 per mouse. All data were represented as the mean ± SD. The differences of **d**, **e** were assessed by one-way analysis of variance (ANOVA). *n* = 10 per group

To confirm the effect of RvD1 on the progression of RA, we treated CIA mice with RvD1 (0, 20, and 100 ng). The result of the mean clinical scores showed that there was no significant difference between the low-dose group and the high-dose group (*p* = 0.370) (Fig. 2b). However, the mean clinical scores of these two groups both exhibited an obvious decrease compared with those of CIA mice, suggesting that RvD1 might slow the development of swelling, erythema, and ankylosis of the hind paws and ankle joints in CIA mice. In addition, histological stain (H&E and toluidine blue, Fig. 2c) showed that RvD1 markedly alleviated joint damage in synovial hyperplasia, invasive pannus, and cartilage erosion in CIA mice, causing a dose-dependent decrease in histological scores (Fig. 2d). Pro-inflammatory factors are crucial for RA progression. For example, IL-6 is deemed to be involved in cartilage damage; IL-1β is considered to be closely related to the onset of RA progression; whereas TNF-α is believed to be connected to the severity of RA [24–26]. Therefore, pro-inflammatory cytokines in mice serum were determined. As shown in Fig. 2e, the concentrations of pro-inflammatory cytokines (IL-6, IL-1β, and TNF-α) in the serum of each treatment group were significantly lower than those of CIA group, which is dose dependent. These results indicated that the inflammatory response could be suppressed by RvD1 in CIA mice. Since CTGF is also an important factor in RA progression, we next tested the expression level of CTGF in CIA mice. The data revealed that RvD1 also markedly decreased the concentration of CTGF in serum of CIA mice (Fig. 2e). Figure 2e shows that the concentration of RvD1 in synovial fluid from mice administered with RvD1 by intravenous injection was higher than in the controls. Taken together, these results indicated that RvD1 is apt to ameliorate joint injury and inflammatory response in the CIA model.

### Suppression effect of RvD1 on RA FLS migration and proliferation

**Fig. 3:**
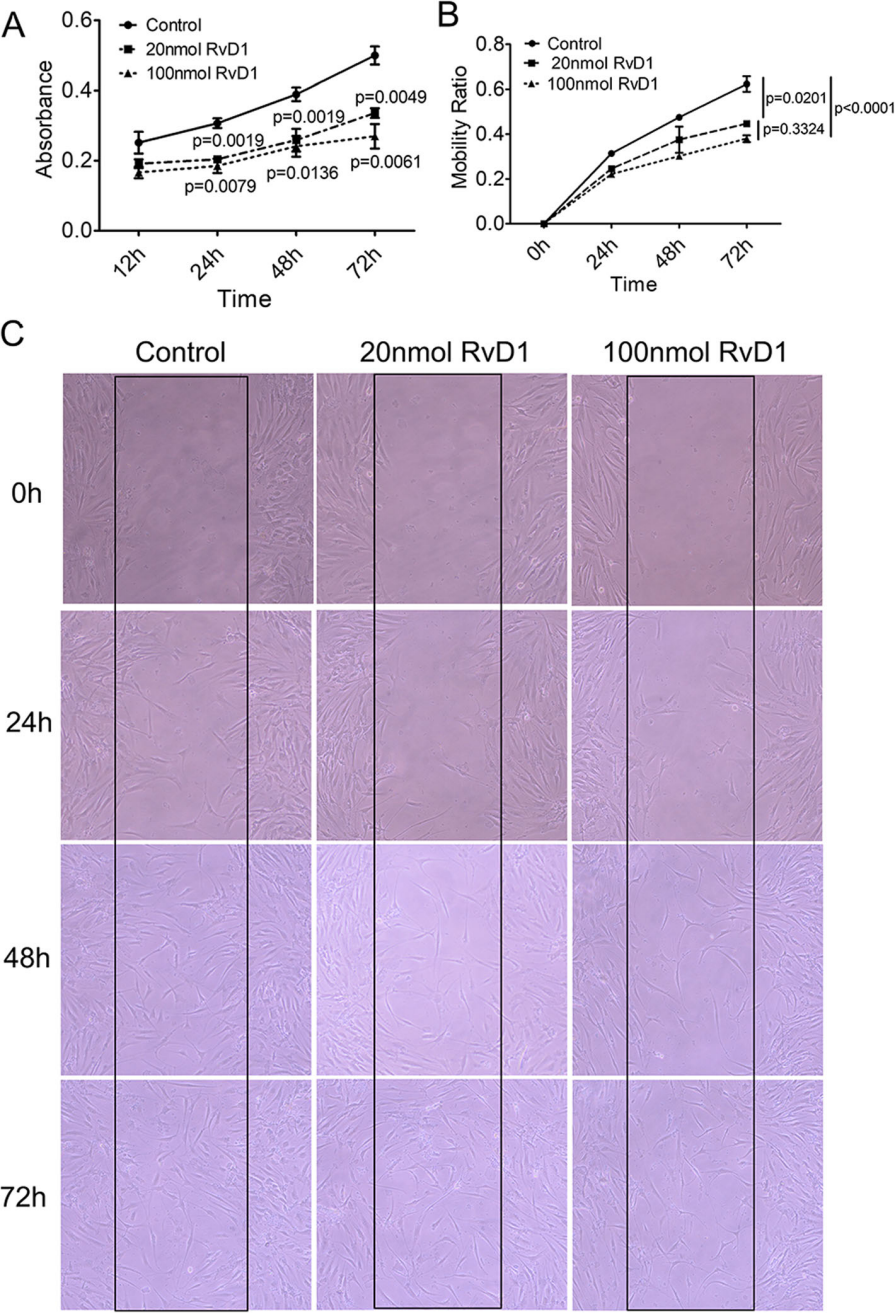
RvD1 suppressed proliferation and migration of RA FLS. **a** MTT assay. The absorbance value at the 490 nm wavelength was measured. The differences among three groups were assessed by one-way analysis of variance. **b**, **c** Scratch migration assay. **b** The mobility ratio of RA FLS. Analysis of variance (ANOVA) of repeated measurement was used to analyze the statistical significance. **c** Images of RA FLS were captured (× 40 magnification). Control, RA FLS were treated without RvD1; 20 nM RvD1, RA FLS were treated 20 nM RvD1; 100 nM RvD1, RA FLS were treated 100 nM RvD1. All data were represented as the mean ± SD

In RA pathogenesis, FLS displayed excessive proliferation, migration to cause cartilage destruction, and acceleration of disease progression [27]. To investigate the effect of RvD1 on FLS proliferation, RA FLS were treated with different concentrations of RvD1 (0, 20, and 100 nM) and proliferation was determined using MTT assay. As shown in Fig. 3a, RA FLS proliferation was obviously inhibited after RvD1 administration for 24–72 h. In addition, the most significant effect emerged at the concentration of 100 nM and the maximum inhibition ratio reached 45% at 72 h. RA FLS migration was detected using scratch migration assay. Similarly, the inhibitory effect of RvD1 on RA FLS migration at the concentration of 100 nM was more significant than that at the concentration of 20 nM, and the maximum inhibitory rate for RA FLS migration was approximately 39% at 72 h (Fig. 3b). Collectively, these results implied that RvD1 could alleviate RA development by suppressing proliferation and migration of FLS.

### Suppression effect of RvD1 on angiogenesis

**Fig. 4:**
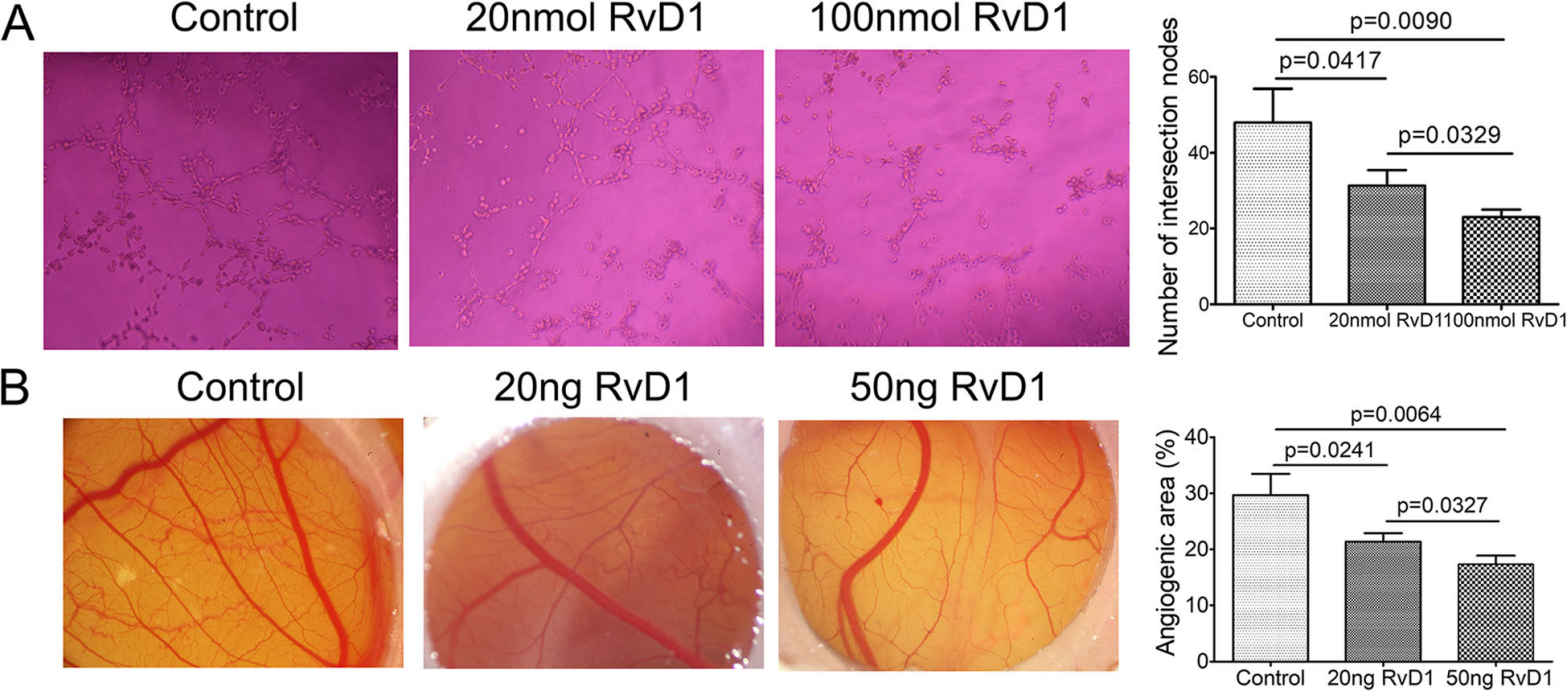
RvD1 decreased angiogenesis. **a** Endothelial tube formation assay. Tube formation were observed and photographed in each well (left panel). The number of intersections among branches of assembled HUVEC networks was calculated in the whole field (right panel). **b** CAM assay. The CAMs were examined and photographed (left panel). The percentage of angiogenic area (right panel) was calculated. *n* = 10 per group. Control, RA FLS were treated without RvD1; 20 nM RvD1, RA FLS were treated 20 nM RvD1; 100 nM RvD1, RA FLS were treated 100 nM RvD1. 20 ng RvD1, CAM was treated with 20 ng RvD1; 50 ng RvD1, CAM was treated with 50 ng RvD1. All data were represented as the mean ± SD. The differences among three groups were assessed by one-way analysis of variance

Angiogenesis is considered to be a critical factor of the pannus formation in the pathogenesis of RA. To assess the effect of RvD1 on angiogenesis in vitro, HUVEC were treated with different concentrations of RvD1 (0, 20, and 100 nM) and analyzed by tube formation assay. According to Fig. 4a, the number of tubes formed by HUVEC treated with RvD1 was obviously less than the control. Moreover, there was not significant statistical difference (*p* = 0.0329) between the number of tubes in the high-concentration (100 nM) group and that in the low-concentration (20 nM) group. On the basis of these results, the effect of RvD1 on the angiogenic activity in vivo was tested using the CAM assay. As shown in Fig. 4b, the number of the small blood vessel in the chorioallantoic membrane exposed to RvD1 for 72 h was significantly lower compared with the control group. These results demonstrated that RvD1 had a potency in terms of anti-angiogenesis.

### RvD1 decreased the expressions of pro-inflammatory cytokines and CTGF in RA FLS

**Fig. 5:**
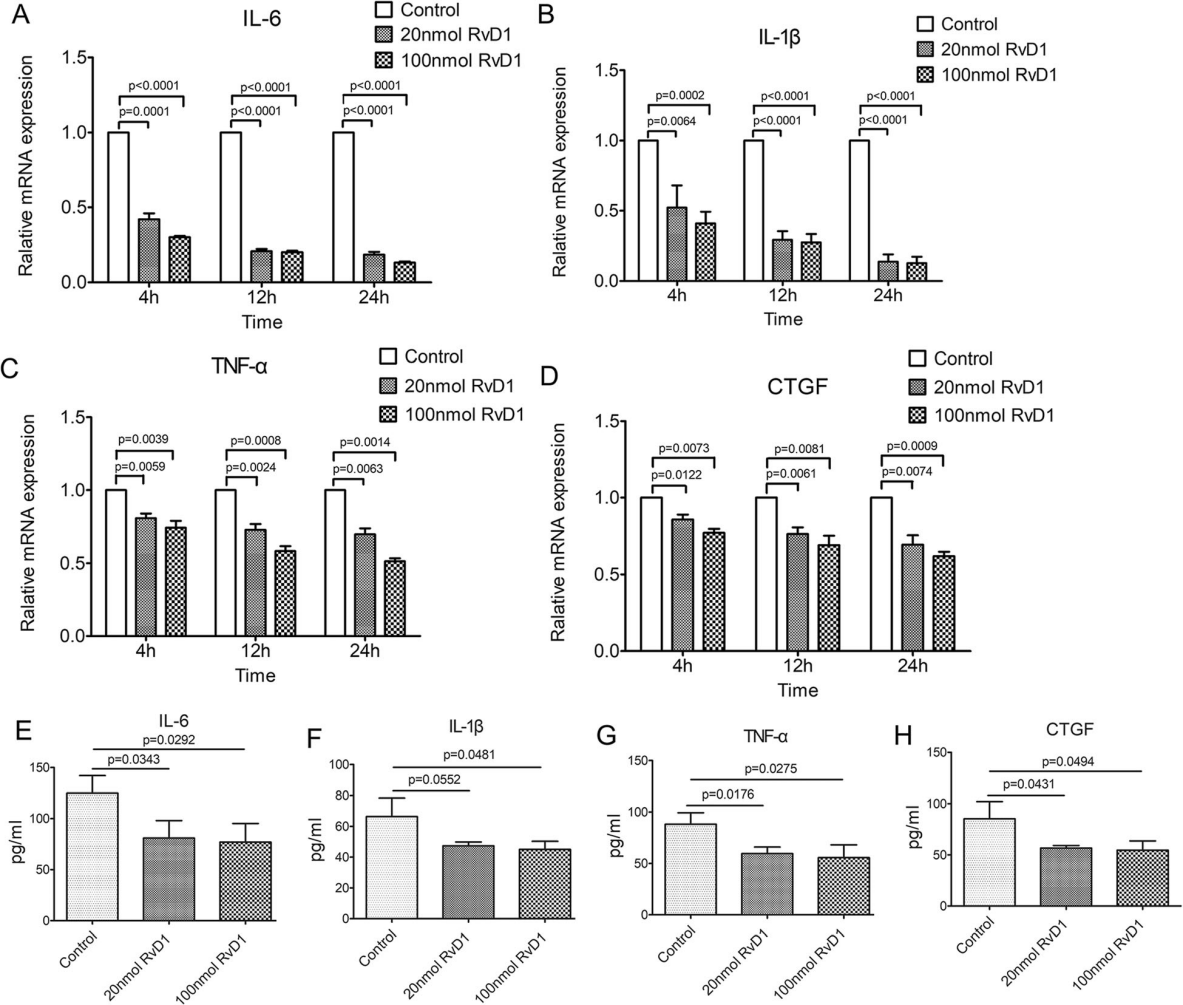
RvD1 decreased the expression of pro-inflammatory cytokines in RA FLS. **a–d** The transcription levels of IL-6 gene, IL-1β gene, TNF-α gene, and CTGF gene in RA FLS treated with RvD1 (0, 20, and 100 nmol) were determined by qRT-PCR. **d–g** The concentrations of IL-6, IL-1β, TNF-α, and CTGF in the supernatant from RA FLS-treated RvD1 were detected by ELISA. Control, RA FLS were treated with PBS; 20 nmol RvD1, RA FLS were treated with 20 nmol RvD1; 100 nmol RvD1, RA FLS were treated with 100 nmol RvD1. All data were represented as the mean ± SD. The differences among three groups were assessed by one-way ANOVA

During the development of RA, FLS become activated and persistently produce inflammatory mediators, including IL-1β, IL-6, and TNF-α, to destroy joint cartilage. The expression profiles of pro-inflammatory cytokines (TNF-α, IL-1β, and IL-6) in RA FLS treated with RvD1 were examined by qRT-PCR. The dissociation curve of all amplified genes showed a single peak, indicating that the amplifications were specific (data not shown). As shown in Fig. 5a–c, TNF-α, IL-1β, and IL-6 genes in RA FLS displayed similar expression profiles: they were significantly downregulated by RvD1. Furthermore, the expression of pro-inflammatory cytokines began to be suppressed when RA FLS were incubated with RvD1 for 4 h, and showed the greatest declines up to 13% (IL-6), 13% (IL-1β), and 49% (TNF-α) at 24 h. Due to the importance of CTGF in cell proliferation and migration, the expression level of CTGF in RA FLS was also measured with qRT-PCR. A maximum decrease (38%) was observed in the RNA level of CTGF after RvD1 treatment (Fig. 5d). Being consistent with these results, the pro-inflammatory cytokines and CTGF were also decreased in the supernatant from RA FLS treated with RvD1 on a protein level (Fig. 5e–h). These data implied that RvD1 could decrease inflammatory response and the expression of CTGF in RA FLS.

### RvD1 decreased the expressions of pro-inflammatory cytokines and CTGF via upregulating miRNA-146-5P in RA FLS

**Fig. 6:**
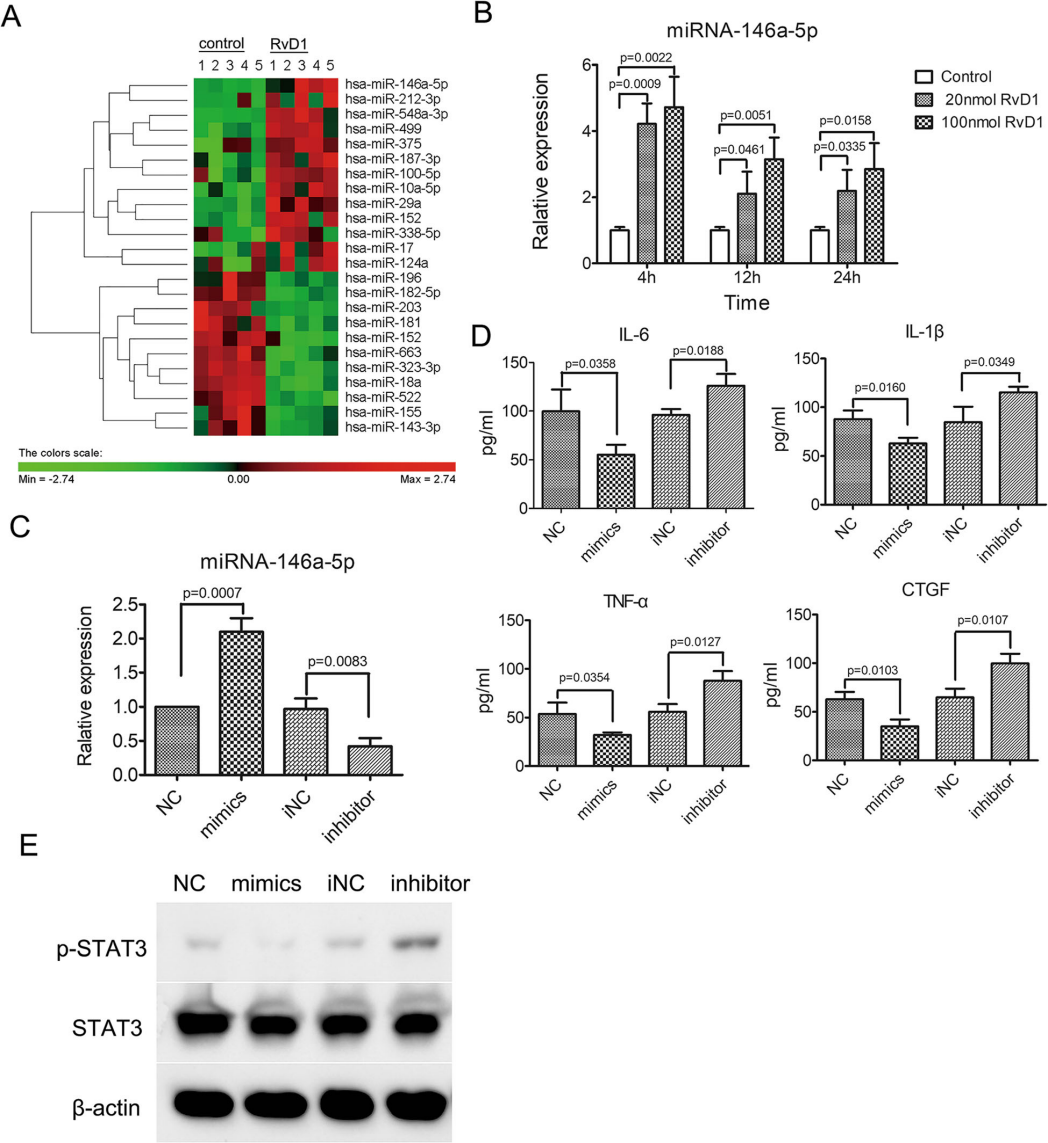
RvD1 decreased the expression of pro-inflammatory cytokines and CTGF in RA FLS through miRNA-146a-5p. **a** The cluster heat map shows 24 miRNAs with expression change fold > 1.5 from miRNA microarray data from the RA FLS-treated RvD1 and control (*p* < 0.05). **b** The expression of miRNA-146a-5p in RA FLS treated with RvD1 (20, 100 nM) was tested by qRT-PCR. **c** The expression of miR146a-5p in RA FLS transfected with miR146a-5p mimics and inhibitor was tested by qRT-PCR. **d** The expression of pro-inflammatory cytokines (TNF-α, IL-1β, and IL-6) and CTGF the supernatant from RA FLS transfected with miR146a-5p mimics and inhibitor were detected by ELISA. **e** The expressions of pSTAT3 and STST3 in RA FLS transfected with miR146a-5p mimics and inhibitor by Western blotting. β-actin was used as loading control. Control, RA FLS were treated with PBS; NC, RA FLS were treated with miR146a-5p negative control; mimics, RA FLS were treated with miR146a-5p mimics; iNC, RA FLS were treated with microR146a-5p inhibitor negative control; inhibitor, RA FLS were treated with miR146a-5p inhibitor. All data were represented as the mean ± SD. The differences among three groups were assessed by one-way ANOVA

To verify the mechanism of RvD1 regulating the expression of pro-inflammatory cytokines and CTGF, the expression profile of microRNA was analyzed by microarray in RA FLS treated with RvD1 and the controls. Figure 6a shows 24 miRNAs with an expression change fold > 1.5 (*p* < 0.05) between RA FLS treated with RvD1 and the controls. Previous research has reported that miRNA-146a-5p is prone to downregulate NF-κB activation through inhibiting translation of mRNA of IL-1 receptor-associated kinase 1 (IRAK1) and TNF receptor-associated factor 6 (TRAF6). Moreover, NF-κB activation is required for various chemokines and cytokines, including IL-6, IL-1β, and so on [7]. Therefore, miRNA-146a-5p was selected for the further qRT-PCR verification experiment. In accordance with the result of microarray, the expression of miR-146a-5p was upregulated dramatically after RvD1 treatment (Fig. 6b), which was also confirmed in CIA mice treated with RvD1 (Fig. 2e). To verify that RvD1 plays an important role via miRNA-146a-5p, the transcription of miRNA-146a-5p was artificially upregulated or downregulated in RA FLS. Compared with that in the control, miRNA-146a-5p was over-expressed in RA FLS transfected with miRNA-146a-5p mimics, whereas it showed the opposite response in RA FLS transfected with miRNA-146a-5p inhibitors (Fig. 6c). Then the mRNA and protein expression levels of pro-inflammatory cytokines and CTGF were determined in RA FLS transfected with miRNA-146a-5p mimics or inhibitor. The results turned out that the inhibition of miRNA-146a-5p caused an increase of the expression of the pro-inflammatory cytokines as well as CTGF in RA FLS; conversely, the over-expression of miRNA-146a-5p reduced the expressions of these genes (Fig. 6d and Fig. S1A-D). These results suggest that miRNA-146a-5p may participate in the course of RvD1 regulating the expressions of pro-inflammatory cytokines and CTGF in RA FLS. To verify the possible mechanism of miRNA-146a-5p decreased CTGF level, the signal of STAT3 was measured by Western blot. The results indicated that miRNA-146a-5p inhibited STAT3 activation (Fig. 6e).

### Suppression effect of miRNA-146a-5p on RA FLS proliferation

**Fig. 7:**
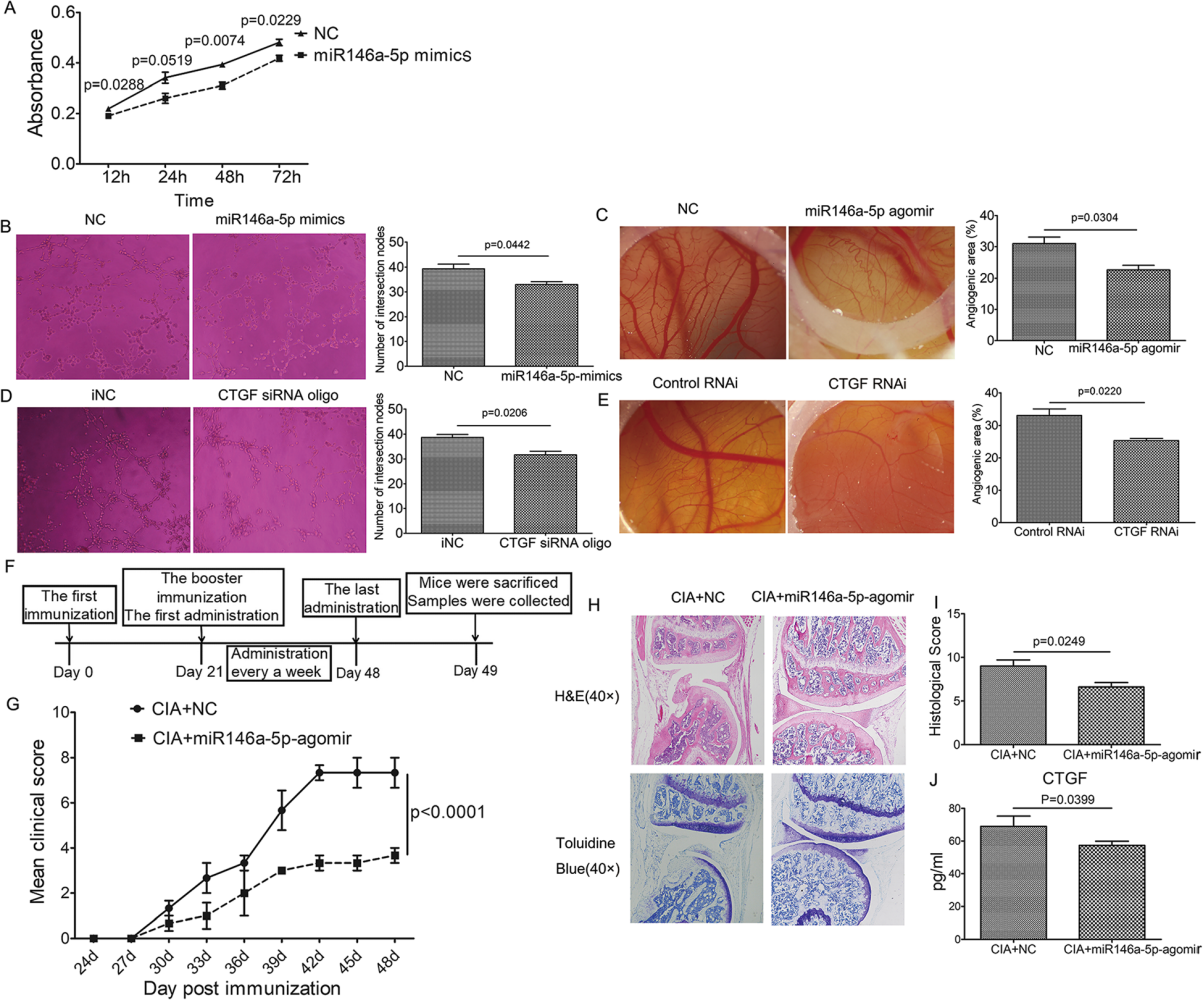
MiRNA-146a-5p inhibited proliferation of RA FLS, angiogenesis and delayed the disease progression on CIA mice, whereas CTGF promoted angiogenesis. **a** MTT assay. RA FLS were transfected with miR146a-5p-mimics and negative control (NC), and the proliferation ability was determined. **b**, **d** Endothelial tube formation assay. HUVEC were transfected with miR146a-5p-mimics negative control (NC), miR146a-5p-mimics, CTGF siRNA negative control (iNC), and CTGF siRNA oligo. Tube formation was photographed (left panel). The number of intersections among branches of assembled HUVEC networks was calculated in the whole field (right panel). **c**, **e** CAM assay. CAM were treated with miR146a-5p-agomir negative control (NC), chick miR146a-5p-agomir, control RNAi, and a lentiviral vector harboring RNAi sequence targeting the CTGF gene (CTGF RNAi). CAMs were examined and photographed (left panel). The percentage of angiogenic area (right panel) was calculated. *n* = 10 per group. **f** The timeline of the miR146a-5p-treated CIA mice experiment. **g** The mean arthritis index scores of mice. Statistical significance was conducted by ANOVA of repeated measurement. **h** H&E and toluidine blue staining of the knee joints of the mice. Semiquantitative scores (**i**) for synovial infiltration, synovial hyperplasia, cartilage damage, and bone damage were assessed. **j** CTGF level in mice synovial fluid was determined by ELISA. *n* = 10 per group. CIA + NC, CIA mice treated with miRNA-146a-5p-agomir negative control; CIA + miR146a-5p-agomir, CIA mice treated with miR146a-5p-agomir. All data were represented as the mean ± SD. Student’s *t* test was used to evaluate the statistical significance

The proliferation ability of RA FLS transfected with miRNA-146a-5p mimics was determined using MTT assay. As shown in Fig. 7a, RA FLS proliferation was obviously inhibited by miRNA-146a-5p during 12–72 h. The result is more evident that the effect of RvD1 on RA is possibly mediated by miRNA-146a-5p.

### Suppression effect of upregulation of miR-146a-5p and downregulation of CTGF on angiogenesis

The effects of miRNA-146a-5p and CTGF on the angiogenesis were detected by tube formation and CAM assays. As shown in Fig. 7b, the number of intersections among branches in HUVEC transfected with miR146a-5p-mimics decreased greatly compared with the control, and the percentage of the angiogenic area in CAM treated with chick miR146a-5p-agomir also decreased significantly (Fig. 7c). Similarly, with CTGF gene interfered, angiogenesis functions were both reduced in HUVEC and CAM (Fig. 7d, e).

### MiRNA-146a-5p attenuated joints damage in CIA mice

Due to the importance of miRNA-146a-5p in the resolution of inflammation and the inhibition on angiogenesis, the CIA model was constructed to verify the effect of miRNA-146a-5p on RA progression. CIA mice treated with mouse miRNA-146a-5p agomir by intra-articular injection displayed an obvious decrease in the mean clinical scores compared with the CIA mice without any treatment (*p* < 0.0001) (Fig. 7g). H&E and toluidine blue staining revealed that miRNA-146a-5p relieved synovial hyperplasia, invasive pannus, and cartilage erosion in the ankle joints of hind paws from CIA mice (Fig. 7h, i). Simultaneously, ELISA results indicated that miRNA-146a-5p agomir induced the decrease of the CTGF level in CIA mice (Fig. 7j). This finding proved further that miRNA-146a-5p is likely to decrease the expression of CTGF in vivo.

## Discussion

Recently, Lima-Garcia et al. found that the aspirin-triggered RvD1 epimer had anti-hyperalgesic and suppression of pro-inflammatory effects in AIA mice through the inhibition of NF-κB activation [13], but the specific mechanism is not yet clear. In the current study, our data revealed that the concentration of RvD1 in serum from RA patients was lower than that in healthy controls. Furthermore, an inverse correlation between the concentrations of CTGF and RvD1 in serum was detected. In addition, we also verified that RvD1 could decrease CTGF expression in RA FLS. CTGF, which is associated with several biological functions such as fibrosis, tumorigenesis, angiogenesis, and endochondral ossification [28, 29], seems to have a close relation with RA. Nozawa et al. demonstrated that CTGF promoted the articular damage by increased osteoclastogenesis in RA patients [30]. Ding et al. found that CTGF could promote articular damage by increased proliferation of FLS in RA [31]. Besides, our previous proteomic research indicated that CTGF expression in FLS from RA patients was remarkably higher than that in the controls [4], and the clinical results revealed that CTGF could be used as a biomarker for the diagnosis of RA. In this study, we demonstrated that CTGF could promote angiogenesis. Therefore, it is natural that we investigate if RvD1 has its effect on angiogenesis.

We found that RvD1 could inhibit angiogenesis by in vitro experiment study. Moreover, we demonstrated that RvD1 could inhibit pannus formation and decrease the levels of pro-inflammation cytokines and CTGF in CIA mice. Considering the role of CTGF in the process of angiogenesis, it came to us that RvD1 could downregulate the expression of CTGF to alleviate RA progression, but the mechanism is still vague.

Therefore, miRNA microarray studies were performed in RA FLS treated with RvD1. Our data revealed that RvD1 upregulated the level of miRNA-146 while synchronously downregulated the level of miRNA-155 and miRNA-181. Based on the *p* value and fold changes, miR-146a-5p was selected for further study. MicroRNA-146a has been widely reported for its multiple roles in the control of the innate and adaptive immune processes and for its oncogenic role in some tumors and arthritis [32]. Boldin et al. found that miRNA-146a-5p played a key role as a molecular brake on inflammation, myeloid cell proliferation, and oncogenic transformation [32]. MiRNA-146a was also known as a major molecular regulator in arthritis. Mice with miRNA-146a deficiency are more likely to develop severe gouty arthritis [33]. What is more, miRNA-146a could inhibit pathogenic bone erosion in inflammatory arthritis [34]. Nakasa et al. affirmed that administration by intravenous injection of miR-146a could prevent joint destruction in CIA mice [35]. We discovered that miRNA-146a could inhibit RA FLS proliferation and angiogenesis. Besides, transfection experiment revealed that over-expression of miRNA-146a-5p in RA FLS significantly decreased inflammatory mediators and CTGF levels. In vivo, we clearly demonstrated that miRNA-146a-5p could inhibit angiogenesis and decreased the level of CTGF to delay RA progression. Taking all the fact into account, we concluded that RvD1 upregulated miRNA-146 to decrease the level of CTGF thus ameliorating RA progression.

As for the mechanism of miR-146a-5p regulating the expression of CTGF, it is predicted by bioinformatics software that CTGF was not the target gene of miRNA-146a-5p, so we concluded that miRNA-146a-5p could not directly bind to CTGF mRNA to decrease CTGF expression. Previous reports and our study have demonstrated that miR-146a-5p can inhibit the producing of IL-6 by suppressing NF-κB signaling pathway. In addition, IL-6 was responsible for STAT3 upregulation [36], and STAT3 signaling activation was required for the expression of CTGF [37]. In our study, the results of Western blotting showed that miRNA-146a-5p inhibited STAT3 activation in the research. Consequently, we speculated that miRNA-146a-5p downregulated IL-6, thereby blocking the expression of STAT3 and finally inhibiting the expression of CTGF. In short, our hypothesis is that miR-146a-5p can downregulate CTGF expression via the IL-6/STAT3 signaling pathway.

Furthermore, miRNA microarray revealed that RvD1 decreased the level of miRNA-155 and miRNA-181 in RA FLS. MiR-155 is also one of the key miRNAs in RA pathogenesis [38]. An increasing number of literatures have supported the concept that miRNA-155 played a key role in keeping Th1/Th2/Th17 balance in the context of autoimmunity [38]. In CIA mice, miRNA-155 played a positive regulatory role in the development of pathogenic Th17 cells [39]. Besides, the homeostasis of Th17/Treg was also regulated by miR-181 in RA. For example, miR-181 was reported to promote the differentiation of Th17 cells [38]. In our study, the expressions of miRNA-155 and miRNA-181 in RA FLS were inhibited by RvD1. Despite the fact that RA FLS are not immune cells, they play a vital role in RA procession. Therefore, the effects of miRNA-155 and miRNA-181 on RA FLS are pending for further study.

## Conclusion

Our current study sheds light on the effect of RvD1 on pannus formation in RA and the underlying mechanism. RvD1 can alleviate RA progression through the upregulation of miRNA-146a-5p to suppress the expression of CTGF and inflammatory mediators, thereby decreasing pannus formation and cartilage damage.

## Supplementary information

Additional file 1: **Fig. S1.** MiRNA-146a-5p decreased the transcription levels of IL-6 gene, IL-1β gene, TNF-α gene and CTGF gene in RA FLS. (A-D) The transcription levels of IL-6 gene, IL-1β gene, TNF-α gene and CTGF gene in RA FLS transfected with miR146a-5p mimics and inhibitor were determined by qRT-PCR. NC, RA FLS were treated with miR146a-5p negative control; mimics, RA FLS were treated with miR146a-5p mimics; iNC, RA FLS were treated with microR146a-5p inhibitor negative control; inhibitor, RA FLS were treated with miR146a-5p inhibitor. All data were represented as the mean ± SD. Student’s t test was used to evaluate the statistical significance.

Additional file 2: **Fig. S2.** RvD1 decreased VEGF level in RA FLS, however it had no effect on the expression of other canonical angiogenic factors (FGF and angiogenin). (A-C) The transcription levels of VEGF gene, FGF gene and angiogenin gene in RA FLS treated with RvD1 (0, 20 and 100 nmol) were determined by qRT-PCR. Control, RA FLS were treated with PBS; RvD1 20 nmol, RA FLS were treated with 20 nmol RvD1; RvD1 100 nmol, RA FLS were treated with 100 nmol RvD1. All data were represented as the mean ± SD. The differences among three groups were assessed by one-way ANOVA.

Additional file 3: **Table 1.** Demographic, clinical, and serological characteristics of blood samples from RA patients and healthy controls.

Additional file 4: **Table 2.** Primers used for real-time PCR analysis.

## Data Availability

The datasets used and/or analyzed during the current study are available from the corresponding author on reasonable request.

## Abbreviations

CAM: Chick chorioallantoic membrane
CIA: Collagen-induced arthritis
CTGF: Connective tissue growth factor
ELISA: Enzyme-linked immunosorbent assay
IL: Interleukin
MTT: 3-(4.5-Dimethylthiazol-2-yl)-2,5-diphenyltetrazolium bromide
PBS: Phosphate-buffered saline
PCR: Polymerase chain reaction
qRT-PCR: Quantitative real-time polymerase chain reaction
RA: Rheumatoid arthritis
RvD1: Resolvin D1
siRNA: Small interfering RNA
STAT: Signal transducer and activator of transcription
TNF: Tumor necrosis factor
UPLC-MS/MS: Ultra-performance liquid chromatography tandem mass spectrometry
