# Supplementary figures and images for: Resolvin D1 suppresses pannus formation via decreasing connective tissue growth factor caused by upregulation of miRNA-146a-5p in rheumatoid arthritis
Source: Arthritis Res Ther. 2020 Mar 27;22:61. doi: 10.1186/s13075-020-2133-2 (PMC7099804; doi:10.1186/s13075-020-2133-2)

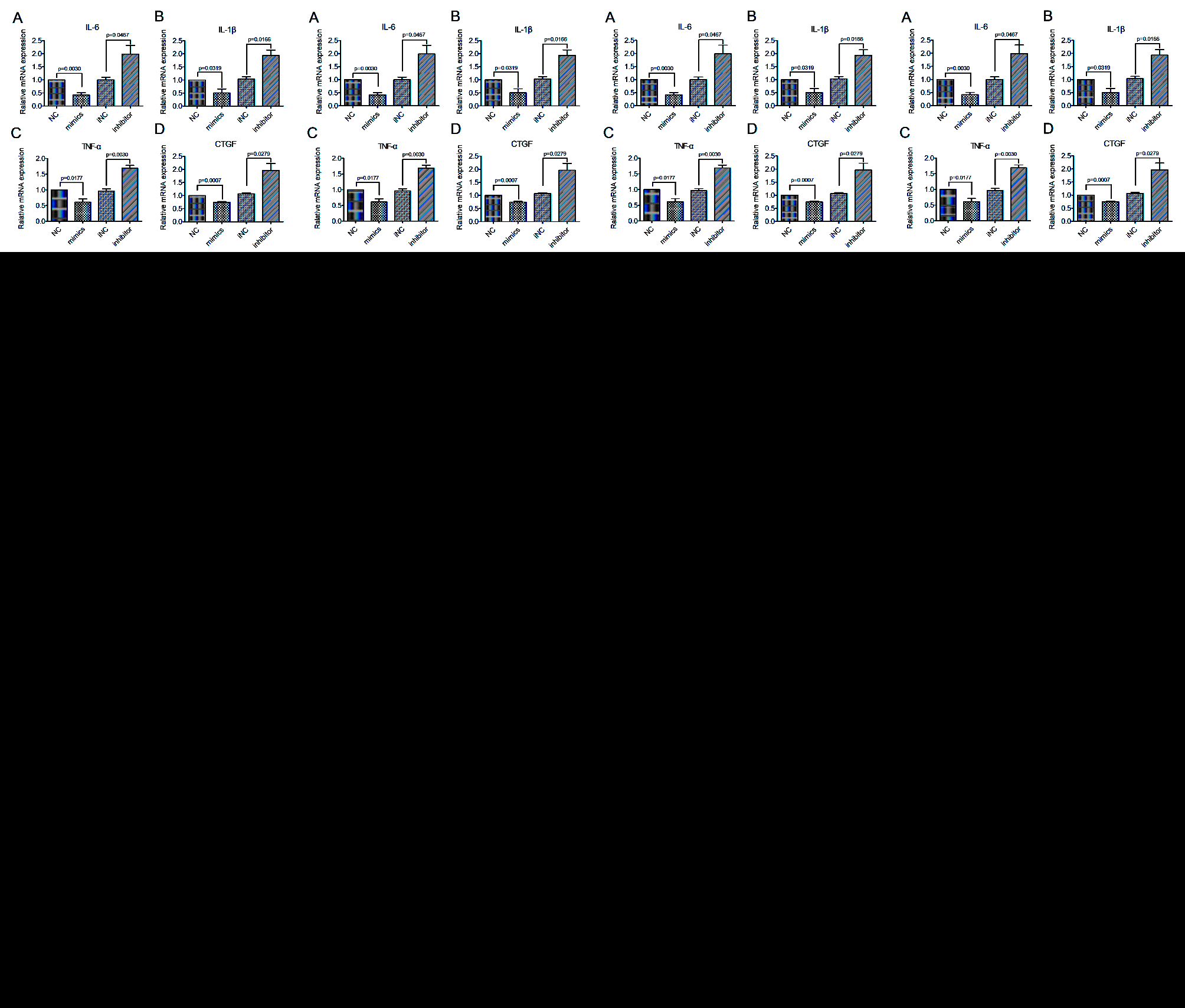

Supplement: Supplementary file 1 — Fig. S1. MiRNA-146a-5p decreased the transcription levels of IL-6 gene, IL-1β gene, TNF-α gene and CTGF gene in RA FLS. (A-D) The transcription levels of IL-6 gene, IL-1β gene, TNF-α gene and CTGF gene in RA FLS transfected with miR146a-5p mimics and inhibitor were determined by qRT-PCR. NC, RA FLS were treated with miR146a-5p negative control; mimics, RA FLS were treated with miR146a-5p mimics; iNC, RA FLS were treated with microR146a-5p inhibitor negative control; inhibitor, RA FLS were treated with miR146a-5p inhibitor. All data were represented as the mean ± SD. Student’s t test was used to evaluate the statistical significance. [file 13075_2020_2133_MOESM1_ESM.tif]

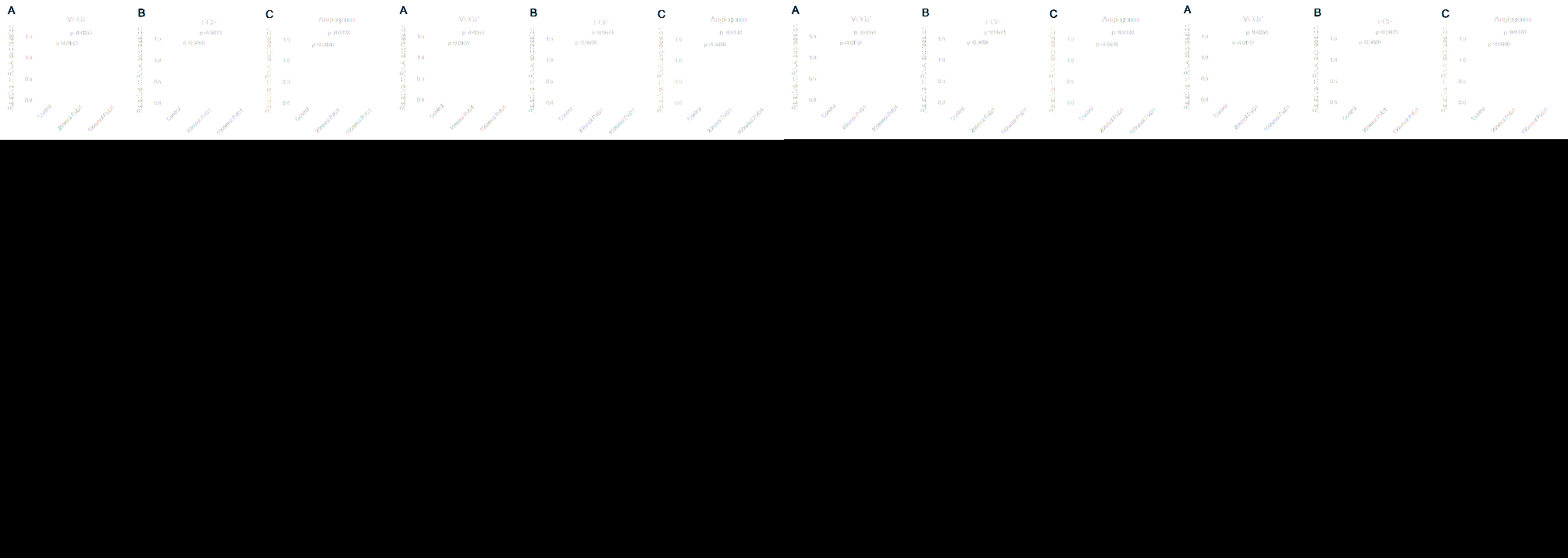

Supplement: Supplementary file 2 — Fig. S2. RvD1 decreased VEGF level in RA FLS, however it had no effect on the expression of other canonical angiogenic factors (FGF and angiogenin). (A-C) The transcription levels of VEGF gene, FGF gene and angiogenin gene in RA FLS treated with RvD1 (0, 20 and 100 nmol) were determined by qRT-PCR. Control, RA FLS were treated with PBS; RvD1 20 nmol, RA FLS were treated with 20 nmol RvD1; RvD1 100 nmol, RA FLS were treated with 100 nmol RvD1. All data were represented as the mean ± SD. The differences among three groups were assessed by one-way ANOVA. [file 13075_2020_2133_MOESM2_ESM.tif]
