# Supplementary material for: Resolvin D1 suppresses pannus formation via decreasing connective tissue growth factor caused by upregulation of miRNA-146a-5p in rheumatoid arthritis
Source: Arthritis Res Ther. 2020 Mar 27;22:61. doi: 10.1186/s13075-020-2133-2 (PMC7099804; doi:10.1186/s13075-020-2133-2)
Supplement: Supplementary file 3 — Table 1. Demographic, clinical, and serological characteristics of blood samples from RA patients and healthy controls. [file 13075_2020_2133_MOESM3_ESM.docx]

Table 1. Demographic, clinical, and serological characteristics of blood samples from RA patients and healthy controls

|  | Normal | RA |
| --- | --- | --- |
| Number,n | 30 | 30 |
| Age (years) | 58 [29, 78] | 55 [24, 80 ] |
| Male/Female, n | 14/16 | 13/17 |
| CRP (mg/L) | NA | 50 [7.23, 170] |
| DAS28 score | NA | 3.98 [2.59, 5.97] |
| ESR (mm/h) | NA | 40 [12, 80] |
| RF positive, n (%) | NA | 22 (73) |
| ACPA positive, n (%) | NA | 23 (77) |
| Duration of disease (years) | NA | 14 [0,30] |

Abbreviations: RA, rheumatoid arthritis; NA, not applicable; CRP, C-reactive protein; ESR, erythrocyte sedimentation rate; RF, rheumatoid factor; ACPA, antibodies directed against citrullinated peptides.
