# Supplementary material for: Resolvin D1 suppresses pannus formation via decreasing connective tissue growth factor caused by upregulation of miRNA-146a-5p in rheumatoid arthritis
Source: Arthritis Res Ther. 2020 Mar 27;22:61. doi: 10.1186/s13075-020-2133-2 (PMC7099804; doi:10.1186/s13075-020-2133-2)
Supplement: Supplementary file 4 — Table 2. Primers used for real-time PCR analysis. [file 13075_2020_2133_MOESM4_ESM.docx]

Table 2. Primers used for real-time PCR analysis.

| Gene | GenBank Accession No. | Forward primer(5′-3′) | Reverse primer (5′-3′) |
| --- | --- | --- | --- |
| IL-1β | NM_000576.2 | AACAGGCTGCTCTGGGATTCTCTTC | CATTGCCACTGTAATAAGCCATCAT |
| IL-6 | NM_001318095 | CTCCAGAACAGATTTGAGAGTAGTG | ATTTGTGGTTGGGTCAGGGGT |
| TNF-α | X02910.1 | CGTCTCCTACCAGACCAAGGTCAAC | GAGGCGTTTGGGAAGGTTGGAT |
| CTGF | CR541734.1 | CTCCCAAAATCTCCAAGCCTATCAA | ACTCCACCGGCAGGGTGGTGGTTCT |
| β-actin | NM_001101.4 | GAGCGGGAAATCGTGCGTGA | CTCGTTGCCGATGGTGATGAC |
| MiRNA146a-5p | NR_029701.1 | CGTGAGAACTGAATTCCATGGGTT | |
